# Amelioration of Behavioural, Biochemical, and Neurophysiological Deficits by Combination of Monosodium Glutamate with Resveratrol/Alpha-Lipoic Acid/Coenzyme Q10 in Rat Model of Cisplatin-Induced Peripheral Neuropathy

**DOI:** 10.1155/2013/565813

**Published:** 2013-12-30

**Authors:** Naini Bhadri, Tejaswi Sanji, Hariprasad Madakasira Guggilla, Rema Razdan

**Affiliations:** Department of Pharmacology, Al-Ameen College of Pharmacy, Bangalore, Karnataka-560027, India

## Abstract

Cisplatin or cis-diamminedichloroplatinum (II) (CDDP) is a cytotoxic chemotherapeutic agent with dose-dependent peripheral neuropathy as a foremost side effect characterised by ataxia, pain, and sensory impairment. Cumulative drug therapy of CDDP is known to produce severe oxidative damage. It mainly targets and accumulates in dorsal root ganglia that in turn cause damage resulting in secondary nerve fibre axonopathy. In the present study, we investigated the neuroprotective effect of the combination of monosodium glutamate (MSG) with three individual antioxidants, that is, resveratrol, alpha-lipoic acid (ALA), and coenzyme Q10 (CoQ10), in cisplatin (2 mg/kg i.p. twice weekly) induced peripheral neuropathy in rats. After 8 weeks of treatment the degree of neuroprotection was determined by measuring behavioral and electrophysiological properties and sciatic nerve lipid peroxidation, as well as glutathione and catalase levels. The results suggested that pretreatment with the combination of MSG (500 mg/kg/day po) with resveratrol (10 mg/kg/day i.p.) or ALA (20 mg/kg/day i.p.) or CoQ10 (10 mg/kg weekly thrice i.p.) exhibited neuroprotective effect. The maximum neuroprotection of MSG was observed in the combination with resveratrol.

## 1. Introduction

Cisplatin is an effective antineoplastic drug widely used in the treatment of malignancies including ovarian, lung, oesophagus, stomach, bladder, and testicular cancers. However, its use is limited by its serious adverse effects including peripheral neuropathy and nephrotoxicity [1]. The reported incidence of neuropathy varies between 24% and 92% [2]. Characteristic features of cisplatin neurotoxicity are mainly ataxia, pain, and sensory impairment. Symptoms are prominent in patients receiving cumulative doses of cisplatin above 300 mg/m^2^ or 500 mg/m^2^ [3]. About 20% of patients have shown poor compliance for the course of cisplatin therapy attributable to sensory neuropathy [4]. The mechanism of cisplatin-induced neuropathy is not completely understood; it is preferentially taken up in to dorsal root ganglia and is believed to affect the stem of the nerves leading to loss of sensory neurons affecting large myelinated sensory nerve fibers [5]. However, data obtained in human studies indicates that cisplatin treatment induces a fall in plasma antioxidant level because of oxidative stress [6] several mechanism including hypoxia, inflammation and free radicals induced apoptosis are thought to be involved. Excessive production of free radicals, such as superoxide anion, hydrogen peroxide, and hydroxyl radicals, and the occurrence of lipid per oxidation due to oxidative stress are associated with cisplatin-induced neuropathy.

Resveratrol (3,5,4′-trihydroxystilbene), a naturally occurring polyphenol found in grapes and red wine, has been reported to have excellent antioxidant activity. It not only prevents free radicals formation but also attenuates their toxicity by inhibiting the lipid per oxidation [7]. It has shown to exert neuroprotective, cancer chemopreventive, anti-inflammatory, and cardioprotective activities [8].

Coenzyme Q10 (CoQ10) or Ubiquinone is an oil-soluble, vitamin-like substance present in almost all eukaryotic cells, primarily in the mitochondria. It is a component of the electron transport chain and participates in aerobic cellular respiration, generating energy in the form of ATP [9]. In recent years research also focused on its antioxidant properties in several cellular compartments [10]. CoQ10 has shown protective effect against neuronal loss in several animal models of neurodegeneration and the ability of CoQ10 to slow disease progression in clinical trials has been considered to explore neuroprotective activity in many of the neurodegenerative diseases [11].

Alpha-lipoic acid or 1,2-dithiolane-3-pentanoic acid (ALA) is a naturally available dithiol compound synthesized enzymatically in the mitochondrion from octanoic acid [12]. ALA reacts with reactive oxygen species such as superoxide radicals, hydroxyl radicals, hypochlorous acid, peroxyl radicals, and singlet oxygen. Administration of ALA has been shown to be beneficial in several oxidative stress models such as ischemia-reperfusion injury, diabetes, cataract formation, HIV activation, neurodegeneration, and radiation injury [13].

Monosodium glutamate (MSG) is the sodium salt of glutamic acid. It is widely used as food additive mainly as flavour enhancer and it is believed to be safe without serious concerns. It contributes an important role in the biosynthesis of several key amino acids [14]. Evidence from clinical and preclinical studies revealed that it is a major oxidative fuel for the gut and that dietary glutamate extensively undergoes first pass metabolism by the intestine. It is an important precursor for synthesis of bioactive molecules, including glutathione, an endogenous antioxidant, and also functions as a key neurotransmitter [15]. Partial investigation of MSG in clinical trial as a neuroprotectant in the early 1980s has shown efficacy in preventing neuropathy induced by vincristine [16]. A new class of glutamate receptors, named metabotropic glutamate receptors (mGluRs) that are coupled to effector systems through GTP proteins (G-proteins), became evident by the majority of most recent research activities. Activation of (mGluRs) subtypes usually regulates many cellular activities in mammalian systems in various experimental models. Activation of (mGluRs) subtypes protected cells against various types of neuronal insults such as traumatic brain injury, stroke, and NMDA excitotoxicity [17]. Interestingly, in recent years, glutamate has been reported to be efficient in preventing neuropathy caused by many cytotoxic drugs in mice, rats, and humans with no or minimal apparent intrinsic toxicity and its low cost and oral availability make it attractive for clinical use as a neuroprotectant [18].

In the present study we further explored its potential in rodents for its neuroprotective activity in presence of other antioxidants, namely, resveratrol, coenzyme Q10, and ALA, in rat model of cisplatin-induced peripheral neuropathy by assessing behavioral, biochemical, and neurophysiological parameters.

## 2. Materials and Methods

### 2.1. Reagents

All reagents and solvents used were of analytical grade. Cisplatin (2 mg/kg i.p. twice weekly) was used to induce peripheral neuropathy by intraperitoneal injection [19]. Aqueous solution of MSG (Himadia Labs, India) was used at 500 mg/kg/day p.o. dose [18]. Resveratrol (Sami Labs, India) solution was freshly prepared using 2% v/v ethanol at a dose of 10 mg/kg/day i.p. [7]. Suspension of ALA (Sami Labs, India) in sterile saline and NaOH was used at a dose 25 mg/kg/day i.p. [20]. Solution of coenzyme Q10 (Himedia Labs, India) was dissolved in 2% v/v ethanol and was used at a dose of 10 mg/kg i.p./day [21]. All solutions were freshly prepared every day before dosing the animals.

### 2.2. Animals

Animal procedures were done in accordance with the Institutional Animal Ethics Committee of CPCSEA. Adult Wistar rats (175–225 g) of both sexes were housed in an aseptic animal room at a temperature of 20–24°C and a humidity of 40–70%, with a 12 h light cycle and 12 fresh air changes per hour in clean paddy husk bedding. All animals were allowed free access to rat chow and water. They were acclimatized for a minimum period of 1 week prior to the beginning of the study.

### 2.3. Induction of Peripheral Neuropathy

Cisplatin (2 mg/kg, i.p. twice weekly) was given for 8 weeks to induce peripheral neuropathy in rats [19]. The dose of cisplatin was decided by the standardization study done previously.

### 2.4. Experimental Design

**Figure 1:**
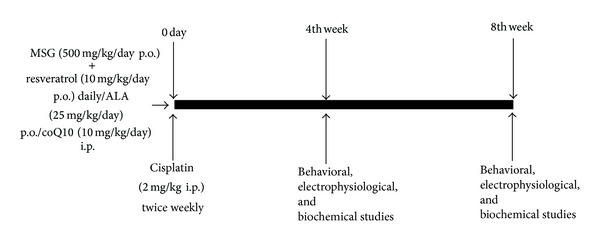
Schematic representation of experimental design.

The animals were divided into five groups (*n* = 6 per group): normal control group (Group 1), cisplatin-induced peripheral neuropathic rats (Group 2), MSG (500 mg/kg/day p.o.) + cisplatin + resveratrol (10 mg/kg/day i.p.) (Group 3), MSG (500 mg/kg/day p.o.) + cisplatin + CoQ10 (10 mg/kg/day i.p.) (Group 4), and MSG (500 mg/kg/day p.o.) + cisplatin + ALA (25 mg/kg/day i.p.) (Group 5) daily for 8 weeks. All parameters under behavioral studies, biochemical estimation, and electrophysiology were evaluated on the completion of the treatment (Figure 1).

### 2.5. Assessment of General Toxicity

Body weight was measured before each administration of cisplatin or vehicle and after administration of drug combination. All rats were examined daily for clinical signs such as piloerection or hind limb weakness and to assess general health.

### 2.6. Behavioral Studies

#### 2.6.1. Thermal Hyperalgesia

Thermal hyperalgesia was assessed by tail immersion test. It was noted with the immersion of terminal part of the tail (1 cm) in water maintained at 50°C. The duration of tail withdrawal reflex or signs of struggle were recorded as response of heat sensation and a cutoff time of 20 s was maintained [22]. Rats were acclimatized to the testing procedure and handled by the investigator during the week before the experiment.

#### 2.6.2. Grip Strength Assays

Grip strength meter was used for evaluating grip strength of animals. Before commencement of experiment the animals were acclimatized by placing them on the instrument for few minutes. Rats were held by the tail above the grid of grip strength meter to an almost horizontal position. The base of the tail was then pulled following the axle of the sensor until it released the grid. The force achieved by the animal was then displayed on the screen and was recorded as newtons or kg units [23].

#### 2.6.3. Rota-Rod Performance Assessment

The rota-rod test is widely used in rodents to assess their “minimal neurological defect” such as impaired motor function (e.g., ataxia) and coordination. The rota-rod unit consists of a rotating rod, 75 mm in diameter, which was divided into four parts by compartmentalization to permit the testing of four rats at a time. Briefly, in a training session, the rats were placed on the rod that was set to 25 rpm and the performance time that each rat was able to remain on the rota-rod was recorded. The rats were subjected to three training trials from 3 h to 4 h intervals on two separate days for acclimatization purposes. In the test session, the rats were placed on the rota-rod and their performance times were recorded [1].

### 2.7. Electrophysiology

#### 2.7.1. Isolation of Sciatic Nerve

Rats were sacrificed by overdose of ketamine/xylazine i.p. Rat's back was shaved and incision was made at L4–L6 spinal segments. The sciatic nerves were surgically exposed from sciatic notch to the gastrocnemius tendon and removed, carefully impregnated on fine filter paper to remove any accompanying blood and soaked for 10 minutes in Ringer-Locke buffer to prevent spontaneous firing of the nerve [24].

#### 2.7.2. Measurement of Nerve Conduction Velocity (NCV)

Rat sciatic nerve having a length of 3 cm–5 cm was prepared and mounted into the nerve chamber consisting of small quantity of phosphate buffer pH 7 to humidify the compartment. First pair of 5 mm spaced electrodes is connected via the stimulator cable (MLA 270) to output 1 and 2 of the power lab unit. The recording cable (MLA 285) was connected to the input 1 of the power lab and microhooks of the same cable to the desired electrode wires depending upon the length of the sciatic nerve. After recording a set of responses latency was calculated using lab chart 7 software. Distance between the stimulating electrode and recording electrode was measured. Nerve conduction velocity (m/sec) was calculated using the following formula:

(1) $$\begin{array}{c} \text{NCV}=\text{Distance}\left(\text{mm}\right)/\text{latency}. \end{array}$$

### 2.8. Biochemical Estimation

#### 2.8.1. Sciatic Nerve Homogenate Preparation

Sciatic nerve samples were rinsed with ice cold saline homogenized in chilled phosphate buffer (pH 7.4) and used for the estimation of lipid per oxidation (MDA levels), reduced glutathione and catalase [25].

#### 2.8.2. Estimation of Lipid per Oxidation

The extent of lipid per oxidation in terms of thiobarbituric acid reactive substances (TBARS) formation was measured according to the method of Esterbauer and Cheeseman. Tissue extracts were mixed separately with 1 mL TCA (20%) and 2 mL TBA (0.67%) and heated for 1 h at 100°C. After cooling, the precipitate was removed by centrifugation. The absorbance of each sample was measured at 535 nm using a blank containing all the reagents except the sample. As 99% TBARS are malondialdehyde (MDA), therefore TBARS concentrations of the samples were calculated using the extinction coefficient of MDA, that is, 1.56 × 10^5^ M^−1^ cm^−1^, and were expressed as *μ*M of malondialdehyde per mg protein [26].

#### 2.8.3. Estimation of Reduced Glutathione

Reduced glutathione was assayed by the method of [27]. Briefly 1.0 mL of sciatic nerve homogenate (10% w/v) was precipitated with 1.0 mL of sulphosalicylic acid (4%). The samples were kept at 40°C for at least 1 h and then subjected to centrifugation at 1200 g for 15 min at 40°C. The assay mixture contained 0.1 mL supernatant, 2.7 mL phosphate buffer (0.1 M, pH 7.4), and 0.2 mL 5,5,dithiobis (2-nitro benzoic acid) (Ellman's reagent, 0.1 mM, pH 8.0) in a total volume of 3.0 mL. The yellow colour developed was read immediately at 412 nm and the reduced GSH levels were expressed as *μ*g/mg protein.

#### 2.8.4. Estimation of Catalase

Catalase was estimated by the method of Aebi [28] which is a quantitative spectroscopic method developed for following the breakdown of H_2_O_2_ at 240 nm in unit time. The sample readings were taken by placing 1 mL of phosphate buffer and 100 *μ*L of tissue homogenate in the reference cuvette and 1 mL of H_2_O_2_ and 100 *μ*L of homogenate in the test cuvette in the spectrophotometer. For each measurement, the reading was taken at 240 nm 1 min after placing the cuvettes in Shimadzu spectrophotometer.

### 2.9. Statistical Analysis

Results were expressed as mean ± S.E.M. The intergroup variation was measured by one-way analysis of variance (ANOVA) followed by Tukey's multiple comparison test. The statistical analysis was done using the Graph pad prism software version 5.0. Probability values *P* < 0.05 were considered to be significant.

## 3. Results

### 3.1. Assessment of General Toxicity

**Figure 2:**
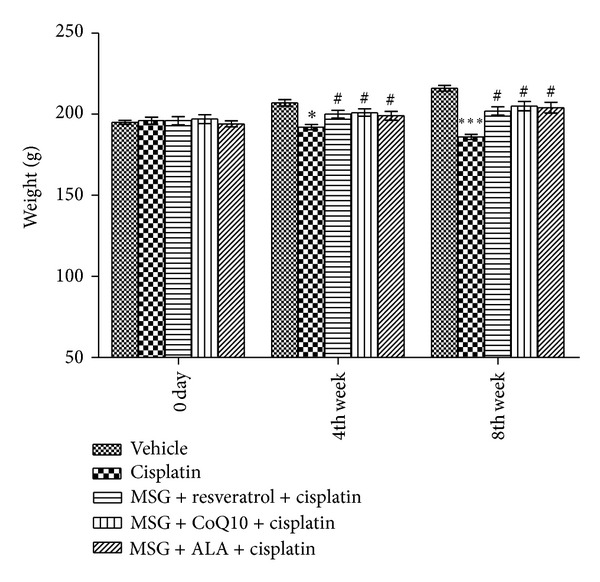
Effect of chronic treatment with combination of MSG and resveratrol/ALA/Co Q10 on body weight. *(*P* < 0.05), ***(*P* < 0.001) versus control group, ^#^(*P* < 0.05) versus cisplatin treated group.

No deterioration in general status was observed; no alterations in the body temperature and no abnormal clinical signs were observed. No rats in the cisplatin control group died during the course of the experiment. Cisplatin treated rats (b.w. 186 ± 1.55 g) showed significant reduction in body weight as compared with the control group (b.w. 216 ± 1.78 g). Treatment with a combination of MSG + resveratrol (b.w. 202 ± 2.55 g), MSG + coenzyme Q10 (b.w. 205 ± 2.9 g), and MSG + ALA (b.w. 204 ± 3.32 g) did not produce any major effect on body weight of the treated rats as compared to the control group (Figure 2).

### 3.2. Behavioral Testing

#### 3.2.1. Effect of Drug Treatment on Nociceptive Threshold in Tail Immersion (Warm Water) Test

**Figure 3:**
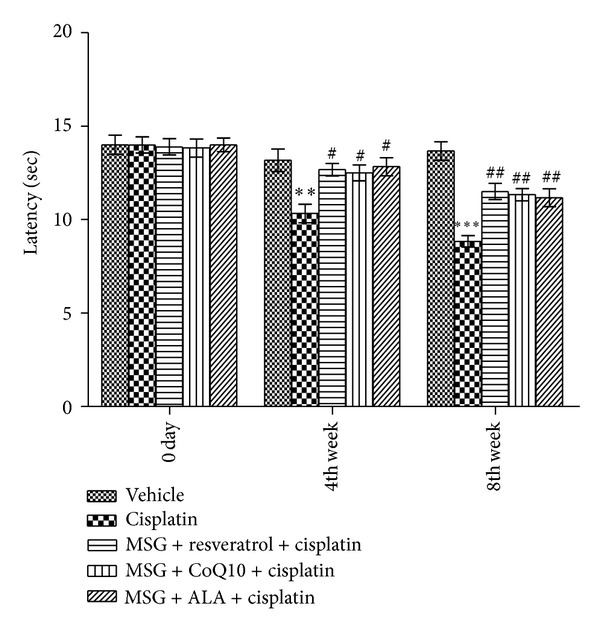
Effect of chronic treatment with combination of MSG and resveratrol/ALA/Co Q10 on thermal hyperalgesia in cisplatin treated rats. **(*P* < 0.001), ***(*P* < 0.001) versus control group, ^#^(*P* < 0.05), ^##^(*P* < 0.01) versus cisplatin treated group.

At the end of the 4th and 8th weeks, cisplatin group exhibited significant decrease in pain threshold from noxious stimuli as compared with control rats (*P* < 0.01). MSG + resveratrol, MSG + Co Q10, and MSG + ALA administration for 8 weeks significantly increased the pain threshold as compared with control neuropathic rats (*P* < 0.01, Figure 3).

#### 3.2.2. Modulation of Muscle Hyperalgesia Using Grip Strength Assay

**Figure 4:**
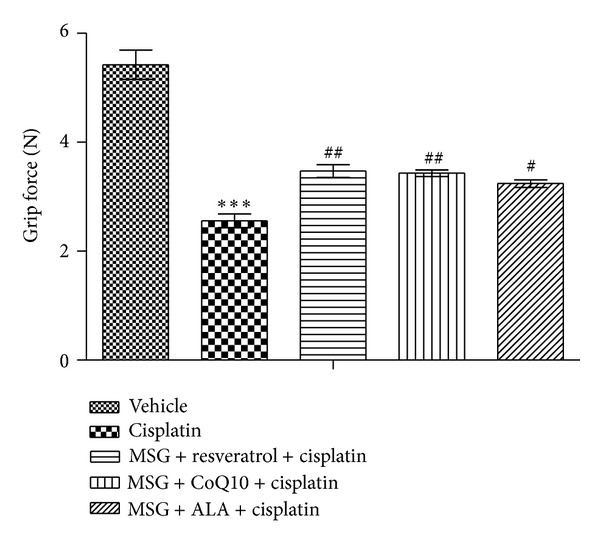
Effect of chronic treatment with combination of MSG and resveratrol/ALA/Co Q10 on grip strength in cisplatin treated rats. ***(*P* < 0.001) versus control group, ^#^(*P* < 0.05), ^##^(*P* < 0.01) versus cisplatin treated group.

Administration of MSG + resveratrol, MSG + CoQ10, and MSG + ALA for 8 weeks significantly improved the latency of grip strength when compared with cisplatin treated rats. Treatment with the combination of MSG + resveratrol and MSG + ALA was the most effective (*P* < 0.01) (Figure 4).

#### 3.2.3. Modulation of Motor Coordination Using Rota-Rod Test

**Figure 5:**
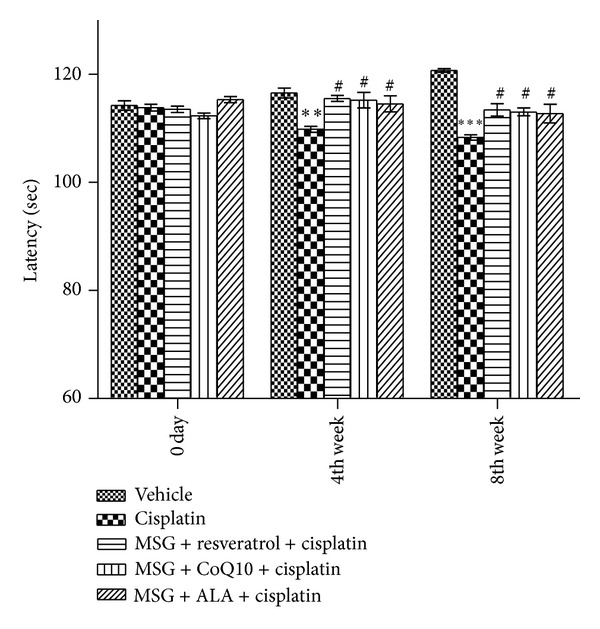
Effect of chronic treatment with combination of MSG and resveratrol/ALA/Co Q10 on motor coordination in cisplatin treated rats. **(*P* < 0.01), ***(*P* < 0.001) versus control group, ^#^(*P* < 0.05), versus cisplatin treated group.

Cisplatin-induced neuropathy impaired motor coordination as evaluated by the walking time on a rotating rod and the number of falls (rota-rod test). Indeed, normal rats were able to balance on the rotating rod for 120.7 ± 0.33 sec as compared with cisplatin treated group (*P* < 0.001). Motor coordination was significantly improved after administration of the combinations of MSG + resveratrol, MSG + CoQ10, and MSG + ALA when compared with cisplatin treated rats. Combination of MSG + resveratrol treatment was the most effective (*P* < 0.05) (Figure 5).

### 3.3. Effect of Cisplatin on Nerve Conduction Velocity

**Figure 6:**
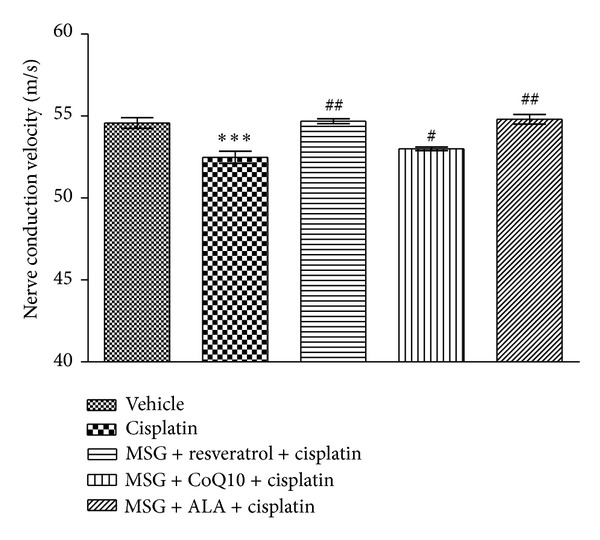
Effect of chronic treatment with combination of MSG and resveratrol/ALA/Co Q10 on nerve conduction velocity in sciatic nerve of cisplatin treated rats. ***(*P* < 0.001) versus control group, ^#^(*P* < 0.05), ^##^(*P* < 0.01) versus cisplatin treated group.

Eight weeks after neuropathy, neuropathic rats showed significant decrease in NCV as compared with the normal group. Treatment with combination of MSG + resveratrol, MSG + CoQ10, and MSG + ALA reversed the nerve conduction in neuropathic rats and significantly improved nerve conduction velocity when compared with cisplatin treated rats. MSG + resveratrol and MSG + ALA treatments were the most effective (Figure 6).

### 3.4. Biochemical Estimation

#### 3.4.1. Effect of Cisplatin-Induced Changes on Lipid per Oxidation

After administration of cisplatin, neuropathic rats showed significant increase in MDA levels in sciatic nerve as compared with the normal group. Treatment with the combination of MSG + resveratrol, MSG + CoQ10, and MSG + ALA at the 4th and 8th weeks showed a significant decrease when compared with cisplatin treated group. MSG + resveratrol treatment was the most effective in reducing the MDA level (Table 1).

#### 3.4.2. Effect of Cisplatin on Reduced Glutathione

Neuropathic rats showed significant decrease in GSH levels in the sciatic nerve as compared with normal group. Treatment with the combination of MSG + resveratrol, MSG + CoQ10, and MSG + ALA showed a significance increase in GSH level. Combination of MSG + resveratrol was the most effective among all combinations (Table 1).

#### 3.4.3. Effect of Cisplatin-Induced Changes on Catalase Activity

Sciatic nerve catalase activity was significantly increased in the 8th week after administration of the combinations of MSG + resveratrol, MSG + CoQ10, and MSG + ALA as compared to cisplatin treated rats in which a lower level of catalase activity was observed (Table 1).

## 4. Discussion

Although cisplatin is an effective antineoplastic drug, its use is limited by its serious adverse effects including peripheral neuropathy and nephrotoxicity [1]. It exhibits its toxic effects on cell by inducing oxidative stress and loss of sensory neurons. In this study the model of cisplatin-induced peripheral neuropathy is used to assess the effectiveness of the few putative combinations of neuroprotective agents, namely, glutamate, resveratrol, ALA, and CoQ10. Rat model is used as it is qualitatively similar to that of humans. It is reported that cisplatin predominantly accumulated in the dorsal root ganglia causing nucleolar damage and also affecting Schwann cells which play a vital role in nerve development and regeneration there by signalling apoptosis [29]. Cisplatin has also been reported to cause ROS generation and causes lipid per oxidation. Recent pieces of evidence suggest that cisplatin reduces peripheral nerve blood supply through potent angiogenic effect which may predispose the nerve damage [3]. Significant loss of body weight, motor coordination abnormality, and a decrease of muscle power could be observed at the 4th week and kept on progressively increasing till the end of the study (8th week). These parameters appeared to be sensitive and reliable to demonstrate neuropathic conditions. Reduction in nerve conduction velocity at the end of treatment confirmed abnormal functions of nerve. Thus, peripheral neuropathy was successfully induced in rats and this model can be used for the screening of potential neuroprotective drugs for the prevention of cisplatin-induced peripheral neuropathy.

In the present study treatment with a combination of MSG (500 mg/kg/day p.o.) with resveratrol (10 mg/kg/day i.p.)/ALA (20 mg/kg/day i.p.)/Co Q10 (10 mg/kg/day i.p.) significantly alleviated cisplatin-induced peripheral neuropathy in rats. Rats treated with the combination exhibited less decrease in body weight, decreased thermal hyperalgesia, improved motor coordination, increased grip strength, improved nerve conduction velocity, and higher levels of antioxidant enzymes compared with cisplatin treated rats.

Resveratrol has been shown to have significant antioxidant and anti-inflammatory properties and found to be a neuroprotective agent against excitotoxicity [30] Reports suggest that resveratrol corrects nerve blood flow deficits due to direct vasodilatation and it has already been shown to produce vasodilatation by both endothelial dependent and endothelium independent pathways [9]. The neuroprotective role of resveratrol in our study could probably be due to its antioxidant activity and improvements in nerve blood flow and restored motor nerve conduction velocity deficits. Nerve blood flow needs to be measured to further strengthen the mechanism of resveratrol in our study. ALA is a potent antioxidant and unlike other antioxidants it is both fat- and water-soluble which works throughout the body. ALA plays a key role in most crucial biochemical functions of mitochondria; thus, it can help with maintaining the mitochondrial membrane potential differential by controlling substrate availability for respiratory chain and regulating the redox state. Several studies provided evidence that ALA is protective in diseases characterized by mitochondrial impairment like diabetic neuropathy [31]. ALA is capable of scavenging a variety of reactive oxygen species. It scavenges hydroxyl radicals and hypochlorous acid and also terminates singlet oxygen. In our study ALA was able to improve the levels of endogenous antioxidants and decreased the lipid per oxidation. These findings could explain the neuroprotective role of ALA.

CoQ10 is a crucial component of the oxidative phosphorylation system in the mitochondria, as it is involved in oxidative phosphorylation of various products of nutrients such as fatty acids and carbohydrates where energy derived from reduced equivalents is converted into ATP to drive cellular machinery and biosynthetic processes [32]. Recent studies explored the new biochemical functions of CoQ10 including the fact that it is considered to be an essential antioxidant, regenerates other antioxidants, stimulates cell growth, and inhibits cell death. CoQ10 protects phospholipids and mitochondrial membrane proteins from per oxidation and protects DNA against oxidative damage there by functions as intracellular antioxidant [33]. In our study CoQ10 was able to reverse the cisplatin-induced oxidative damage by increasing the expression of endogenous antioxidants, preventing lipid per oxidation and thus exhibiting neuroprotective activity in cisplatin treated rats.

Glutamate is involved in the modulation of microtubule functions, which affect intracellular transportation. These processes are important for axons, which require all substances from the cell body for biosynthesis [16]. Our study shows that glutamate in combination with ALA, resveratrol, and Co Q10 has significant neuroprotective effects in cisplatin-induced neuropathy by improving endogenous antioxidant levels and probably by exerting an indirect effect on microtubules after interacting with a receptor found only on neural cells; other possible links in this pathway include local growth factor release or alteration of intracellular calmodulin and ATP which have been shown to be regulators of microtubule formation and stability [18]. However, the neuroprotective effect of glutamate can be further explained and explored by measuring the calmodulin and ATP.

## 5. Conclusion

In conclusion, we have demonstrated that MSG, resveratrol, ALA, and CoQ10 combination protected against cisplatin-induced neurotoxicity by increasing endogenous antioxidants, preventing lipid per oxidation and improving NCV. Thus, these results would encourage future clinical trials of these agents in cancer patients to whom coadministration of neuroprotectants is required to prevent neurotoxicity.

## Figures and Tables

**Table 1:** Effect of chronic treatment with combination of MSG and resveratrol/ALA/Co Q10 on levels of MDA (a marker of lipid peroxidation), reduced glutathion, and catalase in sciatic nerve of cisplatin treated rats.

SL. NO	Group	MDA (*µ*M/mg protein)	GSH (*µ*g/mg protein)	Catalase (U/mg protein)
1	Vehicle	1.52 ± 0.09	70.34 ± 0.33	0.106 ± 0.010
2	Cisplatin	2.76 ± 0.19***	57.66 ± 0.69***	0.051 ± 0.004***
3	Cisplatin + MSG + Res	2.12 ± 0.08^##^	61.72 ± 1.19^##^	0.091 ± 0.006^##^
4	Cisplatin + MSG + Co Q10	2.22 ± 0.04^#^	60.63 ± 0.47^#^	0.088 ± 0.008^#^
5	Cisplatin + MSG + ALA	2.32 ± 0.02^#^	60.76 ± 0.49^#^	0.095 ± 0.010^##^

***(*P* < 0.001) versus control group, ^#^(*P* < 0.05), ^##^(*P* < 0.01) versus cisplatin treated group.
